# A Comprehensive Review on the Application of Artificial Intelligence for Predicting Postsurgical Recurrence Risk in Early‐Stage Non‐Small Cell Lung Cancer Using Computed Tomography, Positron Emission Tomography, and Clinical Data

**DOI:** 10.1002/jmrs.860

**Published:** 2025-01-23

**Authors:** Ghazal Mehri‐kakavand, Sibusiso Mdletshe, Alan Wang

**Affiliations:** ^1^ Department of Anatomy and Medical Imaging, Faculty of Medical and Health Sciences University of Auckland Auckland New Zealand; ^2^ Auckland Bioengineering Institute The University of Auckland Auckland New Zealand; ^3^ Centre for Brain Research The University of Auckland Auckland New Zealand; ^4^ Matai Medical Research Institute Gisborne New Zealand; ^5^ Medical Imaging Research Centre The University of Auckland Auckland New Zealand; ^6^ Centre for Co‐Created Ageing Research The University of Auckland Auckland New Zealand

**Keywords:** artificial intelligence, non‐small cell lung cancer, PET/CT, radiomics, recurrence prediction

## Abstract

**Introduction:**

Non‐small cell lung cancer (NSCLC) is the leading cause of cancer‐related mortality worldwide. Despite advancements in early detection and treatment, postsurgical recurrence remains a significant challenge, occurring in 30%–55% of patients within 5 years after surgery. This review analysed existing studies on the utilisation of artificial intelligence (AI), incorporating CT, PET, and clinical data, for predicting recurrence risk in early‐stage NSCLCs.

**Methods:**

A literature search was conducted across multiple databases, focusing on studies published between 2018 and 2024 that employed radiomics, machine learning, and deep learning based on preoperative positron emission tomography (PET), computed tomography (CT), and PET/CT, with or without clinical data integration. Sixteen studies met the inclusion criteria and were assessed for methodological quality using the METhodological RadiomICs Score (METRICS).

**Results:**

The reviewed studies demonstrated the potential of radiomics and AI models in predicting postoperative recurrence risk. Various approaches showed promising results, including handcrafted radiomics features, deep learning models, and multimodal models combining different imaging modalities with clinical data. However, several challenges and limitations were identified, such as small sample sizes, lack of external validation, interpretability issues, and the need for effective multimodal imaging techniques.

**Conclusions:**

Future research should focus on conducting larger, prospective, multicentre studies, improving data integration and interpretability, enhancing the fusion of imaging modalities, assessing clinical utility, standardising methodologies, and fostering collaboration among researchers and institutions. Addressing these aspects will advance the development of robust and generalizable AI models for predicting postsurgical recurrence risk in early‐stage NSCLC, ultimately improving patient care and outcomes.

## Graphical abstract

Our review provides a comprehensive and up‐to‐date analysis of AI techniques, including radiomics, machine learning, and deep learning, for predicting postoperative recurrence risk in early‐stage NSCLC. We critically evaluated 16 high‐quality studies published between 2018 and 2024, focusing on the most recent and innovative approaches. Our findings identify promising areas for future research, emphasise the importance of multimodal approaches, and highlight challenges that need to be addressed for clinical integration. The results have significant implications for advancing prognostic assessment and personalised treatment strategies for NSCLC patients.

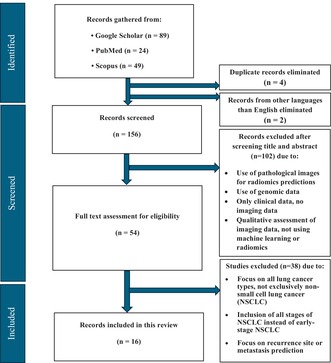

## 1 Introduction

### 1.1 Background

Lung cancer remains the most common cause of cancer‐related deaths globally, surpassing colorectal, breast, brain, and prostate cancers [1]. In 2022, lung cancer emerged as the most prevalent malignancy, accounting for an estimated 2.5 million newly diagnosed cases, which constituted 12.4% of the global cancer burden. Moreover, it was the primary contributor to cancer‐related mortality, with approximately 1.8 million deaths attributed to the disease, representing 18.7% of all cancer fatalities worldwide [2]. Lung cancer is categorised into two main types: small cell lung cancer (SCLC) and non‐small cell lung cancer (NSCLC), with NSCLC comprising about 85% of all cases [3, 4]. Although chemotherapy and radiotherapy have seen recent advancements, surgery remains the primary treatment option for early‐stage non‐small cell lung cancer [5]. Recent advancements in low‐dose computed tomography (LDCT) screening for early‐stage NSCLC have led to early diagnosis with a potential for cure. This is crucial because early detection allows for prompt treatment, ultimately resulting in reduced mortality rates [6]. Despite the potential for diagnosing the tumour and resecting it in the early stage, recurrence affects 30%–55% of patients within 5 years following surgery, resulting in decreased quality of life and, in severe cases, mortality [7, 8].

Patients with surgical interventions characterised by incomplete tumour excision, encompassing residual macroscopic lesions or microscopically positive margins, are typically considered candidates for adjuvant therapeutic interventions. Empirical evidence demonstrates that despite comprehensive post‐surgical adjuvant treatments, individuals with microscopically compromised resection margins persistently exhibit substantially diminished survival outcomes compared to patients achieving complete tumour resection [9]. Complete surgical excision also does not guarantee uniform clinical outcomes among NSCLC patients, with significant inter‐patient prognostic divergences observed [10]. Despite efforts to prevent recurrence through adjuvant therapy, such as radiotherapy and chemotherapy [11], survival advantages are not consistently observed [11, 12]. This is because, on the one hand, the toxicity of chemotherapy is a leading cause of post‐surgery mortality [9], and on the other hand, the prognosis in each patient is influenced by multiple factors, including disease stage, molecular composition, histopathological features, patient age, gender, baseline functional status, and concurrent health issues [13]. As a result, the use of adjuvant chemotherapy in NSCLC is controversial, and there is a need to identify the best candidates and the most effective chemotherapy regimens. Thus, this underscores the importance of a personalised treatment plan for every patient [14]. Innovating novel prognostic methodologies is imperative for effectively managing NSCLC patients, especially in choosing optimal treatment strategies and forecasting prognosis [15].

The tumour node metastasis (TNM) staging system remains the predominant scientific benchmark for predicting patient outcomes [16, 17]. Achieving accurate lung cancer staging demands imaging modalities capable of characterising the primary tumour (T) and providing a precise assessment of both local nodal (N) and distant tumour spread (M) [18].

### 1.2 Imaging in NSCLC

Advanced imaging technologies, specifically computed tomography (CT) and positron emission tomography (PET) have emerged as the most reliable methodologies for stratifying lung cancer progression and estimating recurrence probabilities [19]. Utilising these imaging modalities, radiologists analyse and stage the disease. By correlating radiological observations with molecular and histopathological insights, medical professionals can plan treatment approaches [20]. Nonetheless, significant heterogeneity in patient survival outcomes remains a prominent clinical observation [21]. Tumours classified under the same stage and histology can exhibit distinct biological characteristics, including driver mutations, variations in proteomic profiles, genomic diversity, and unique microenvironments. These factors influence tumour behaviour, aggressiveness, and response to treatment [22]. Hence, an exact estimation of recurrence risk following surgery in NSCLC patients at diagnosis could prove vital in guiding tailored therapies, averting both excessive and insufficient treatment of NSCLC patients [23].

### 1.3 Artificial Intelligence in the Estimation of NSCLC Recurrence

Several approaches have been considered to enhance the estimation of recurrence. For example, artificial intelligence (AI), which refers to machine (computer) intelligence, resembles human‐like cognitive functions, meaning that it learns patterns and insights directly from data to identify, interpret, and autonomously address similar situations [24]. The application of AI in medical image interpretation has introduced the potential for more automated and precise evaluations, which may help reduce physician workloads and improve diagnostic accuracy and turnaround times. However, the true effectiveness of these algorithms is complex to assess and will require further research and clinical validation in the coming years [25].

Radiomics, machine learning (ML), and deep learning (DL) are subcategories of AI, and it is crucial to recognise that they are not isolated domains but rather closely interconnected disciplines [26]. Table 1 summarises key AI concepts with brief definitions, main features, and associated challenges for better comprehension. Adoption of these techniques could enhance diagnostic and prognostic assessments, reduce subjective biases, and improve data consistency [27].

**TABLE 1:** Key definitions, features, and challenges of radiomics, machine learning, deep learning, and convolutional neural networks in AI applications for medical imaging.

Term	Definition	Key Features	Challenges
Radiomics	Extraction of quantifiable features from medical images to characterise tissue properties	Captures pixel intensity, shape, texture, and mesoscopic details	Limited information from single modalities; human bias in interpretation
Machine learning (ML)	A subset of AI focused on algorithms that detect patterns in data, learn from them, and make decisions without explicit programming	Learns from data, adapts to new patterns, and evolves through experience	Requires well‐curated data and can be prone to overfitting with small datasets
Deep learning (DL)	A specialised subset of ML using neural networks with multiple layers to model complex patterns and large datasets	Automates feature extraction, handles massive datasets, and enhances precision	‘Black box’ nature; limited interpretability of results; requires significant computational resources
Convolutional Neural Networks (CNNs)	A type of deep learning algorithm designed to analyse visual data, such as medical images	Automatically identifies complex patterns in imaging data; excellent for tasks like tumour detection	‘Black box’ issue; high computational cost; may require large, annotated datasets for training

For instance, radiomics‐based models have been used to predict recurrence by extracting high‐dimensional features from PET and CT images and linking them to tumour heterogeneity [10, 17, 28, 29]. These models demonstrated potential in identifying early‐stage NSCLC patients at a high risk of recurrence, but their generalizability was limited due to variations in imaging protocols and feature extraction methods. Similarly, machine learning algorithms, such as random forest classifiers, have been employed to integrate clinical and imaging data, showing improved predictive accuracy compared to traditional methods [17, 29, 30, 31]. Deep learning models, including convolutional neural networks (CNNs), have shown promise by automatically learning features from imaging data without manual engineering [7, 23, 32, 33]. However, these models face the ‘black box’ challenge, making it difficult for clinicians to interpret predictions and trust the outcomes [34].

The clinical implications of AI‐based recurrence estimation are substantial. By identifying patients at higher risk of recurrence, AI can inform personalised treatment plans, allowing for closer monitoring, earlier intervention, or adjustments in therapy intensity [35]. This could lead to improved treatment outcomes, reduced recurrence rates, and better long‐term survival for NSCLC patients [36]. Furthermore, AI can optimise resource allocation in clinical settings by guiding decisions on which patients require intensive follow‐up or additional testing, ultimately improving efficiency and reducing healthcare costs [37].

### 1.4 Rationale

The primary objectives of this review were threefold: (1) to conduct a comprehensive analysis and critical appraisal of the existing literature on the utilisation of artificial intelligence (AI) techniques for predicting the risk of postoperative recurrence in patients with early‐stage non‐small cell lung cancer (NSCLC), with a particular emphasis on models leveraging CT and PET imaging data; (2) to examine the implementation of various AI models and identify promising avenues for future research and investigation; and (3) to evaluate the impact of multimodality approaches, combining CT, PET, and clinical factors, on the performance and predictive accuracy of these AI‐based prognostic models.

## 2 Methods

### 2.1 Study Design and Methodology

#### 2.1.1 Research Design

This article presented a literature review study that aimed to map out the current literature focusing on the utilisation of AI in predicting early‐stage NSCLC postoperative recurrence risk and explored the conceptual boundaries of the topic while indicating the volume of literature available. Relevant journal articles in databases were identified by searching using specific keywords. Overall, the intention of this review was to provide an overview of the current state of research, identify key findings and methodological approaches, and highlight promising areas for future investigation in the application of AI for predicting postoperative recurrence risk in early‐stage NSCLC patients. While this review followed a structured narrative review approach, it did not meet the criteria for a full systematic review due to the practical constraints of being part of a PhD project. Despite this, a rigorous methodology was employed to critically analyse the literature within the available scope and resources.

#### 2.1.2 Methodology

To identify relevant literature, a comprehensive search was conducted using the following search terms: (NSCLC OR “non‐small cell lung cancer”) AND (“Recurrence Prediction” OR “Relapse Prediction” OR “Prognosis Prediction”) AND (“Artificial Intelligence” OR “AI” OR “Machine learning” OR “Deep learning” OR “Neural network” OR “Radiomics”) AND (“Imaging biomarkers” OR “PET” OR “CT” OR “PET/CT”). The databases Google Scholar, PubMed, and Scopus were chosen for their broad coverage of biomedical, imaging, and AI research.

**FIGURE 1:**
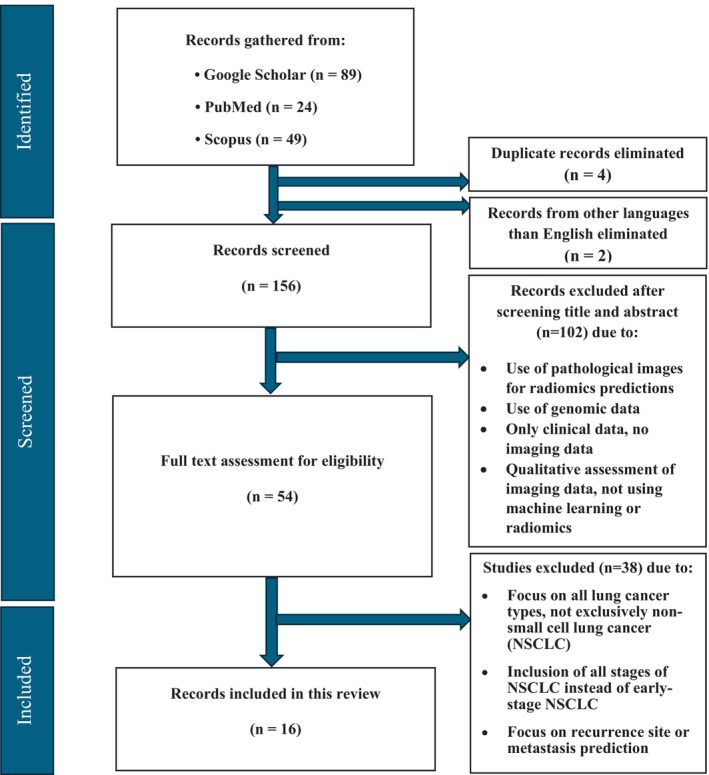
PRISMA flow diagram of study selection.

Figure 1 depicts the PRISMA workflow of study selection [38]. Eligibility criteria included patients with early‐stage non‐small cell lung cancer (NSCLC), undergoing “surgery” as treatment, recurrence prediction using radiomics, machine learning, deep learning, or traditional statistical analysis based on preoperative PET, CT, PET/CT, with or without integrating with clinical data. Exclusion criteria consisted of studies not written in English, non‐original articles (such as review articles and conference abstracts), those integrating genomics and pathological images, and studies focusing on recurrence site or metastasis prediction. Considering the substantial research conducted in radiomics, ML, and DL, this study limited its search to articles published between 2018 and 2024 to concentrate solely on the most recent state‐of‐the‐art studies.

### 2.2 Data Extraction

Data that were extracted from studies included first author, publication year, size of the patient cohort, number of the cohorts, retrospective or prospective, stage of NSCLC, type of treatment, prognosis outcome, imaging modality with or without clinical data, plain or contrast CT, models, baseline model for comparison, segmentation methods/tools, feature extraction methods/tools, feature selection methods/tools, model with the best performance, features that best define the prognosis, the existence of external validation, study aims, and key findings. The data was summarised into an Excel spreadsheet.

### 2.3 Quality Assessment Method

To assess the methodological quality of the included studies in this review, we utilised the METhodological RadiomICs Score (METRICS), a recently developed quality scoring tool endorsed by the European Society of Medical Imaging Informatics (EuSoMII) [39]. To calculate the METRICS score for each included study, we used a web application that has been developed to help with the calculation of the METRICS score (https://metricsscore.github.io/metrics/METRICS.html). The METRICS tool includes 30 items organised into nine categories: study design (Item #1, 2, and 3), imaging data (Item #4, 5, 6, and 7), segmentation (Item #8, 9, and 10), image processing and feature extraction (Item #11, 12, and 13), feature processing (Item #14, 15, 16, and 17), preparation for modelling (Item #18 and 19), metrics and comparison (Item #20, 21, 22, 23, 24, and 25), testing (Item #26 and 27), and open science (Item#28, 29, and 30). Each item is assigned a weight based on the expert panel's assessment of its relative importance in radiomics research. The tool accounts for different radiomics pipeline designs, including handcrafted radiomics, deep radiomics, and end‐to‐end DL approaches, by incorporating conditional items.

## 3 Results

### 3.1 Data Extraction From the Selected Literature

The study selection process is outlined in the PRISMA flowchart (Figure 1). A comprehensive search across Google Scholar, PubMed, and Scopus identified 162 articles. After removing four duplicates and two non‐English articles, 156 remained for screening. Titles and abstracts were reviewed by the first author, with results confirmed by two co‐authors. Uncertain cases were resolved collaboratively. During the screening, 102 articles were excluded for relying on pathological images and genomic data, focusing solely on clinical data, using qualitative imaging assessments without machine learning or radiomics. This left 54 articles for full‐text review. The first author conducted the review and data extraction, with results verified by the co‐authors. After resolving discrepancies, 38 articles were excluded for addressing all lung cancer types or all stages instead of early‐stage NSCLC, focusing on recurrence site or metastasis prediction rather than overall recurrence risk prediction. Ultimately, 16 articles met all inclusion criteria and were included in this review [7, 17, 19, 23, 28, 29, 30, 31, 32, 33, 34, 40, 41, 42, 43, 44]. The METRICS score for each study is summarised in Table S1.

### 3.2 Patient Demographics and Study Features

#### 3.2.1 Modalities

The characteristics of each of these 16 studies are presented in Table 2. All of them were published from 2018 to 2024. Apart from one study [45], others were retrospective in nature. Four studies used just CT images (4/16, 25%) [23, 30, 32, 45], of which one used contrast CT images [32]. Eight studies used both CT images and clinical data (8/16, 50%) [7, 29, 31, 33, 40, 41, 42, 43], of which one of them used contrast images with clinical data [40]. One study used radiomics‐based PET images (1/16, 6.25%) [28]. Three studies used PET, CT, and clinical data (3/16, 18.75%) [10, 17, 19].

**TABLE 2:** Overview of the included studies.

Study ID and Year	Data size, no. of cohorts, prognosis outcome.	Image/Clinical modalities	Model (RM (HCR)/DLR/SA/ML/DL)	Baseline	Segmentation	Feature extraction	Feature selection	Best performance model	Aim	Key findings	Metric score
Hwan‐ho Cho [45] 2021	617, 1, PP and OS	CT	HCR + DLR (Yolo v3, Dense, and VGG)	Compare DRS with RRS	Manual (MATLAB)	Pyradiomics	Cox‐LASSO	Yolo model, Hazard ratio = 6.5362, *p*‐value = 0.0022, C‐index = 0.7696	Build a prognostic model by correlating DL features with radiomics to increase the interpretability of DL features an offset sample size limitation	Radiomics‐guided Yolo model improved prognosis stratification	88.8% (E)
Samantha Bove [7] 2023	144, 1, RP	CT + Clinical	DLR (AlexNet, ResNet152V2, InceptionV3) for feature extraction + SVM as classifier	Different CNN models + different ROI sizes	Manual (ePAD software)	Pre‐trained CNNs	Variance filtering and area under ROC curve threshold	AlexNet CNN on CROP 20 ROI, AUC = 0.83, Acc = 0.79, Sens = 0.80, Spec = 0.78	To investigate the predictive power of the peritumoral region in predicting recurrence	Including peritumoral region improved prediction, AlexNet performed best	50.5% (M)
Annarita Fanizzi [23] 2023	144, 1, RP	CT	DLR (ViTs, PVTs, STs, and CNNs)	CNNs (ResNet50, DenseNet201, InceptionV3)	Manual (ePAD software)	Pre‐trained DL models	NA	CNN (InceptionV3), AUC = 0.91, Acc = 0.89, Sens = 0.85, Spec = 0.90, Precision = 0.78	Compare vision transformers and CNNs in predicting NSCLC recurrence	While CNNs (InceptionV3) performed best, vision transformers like PVT‐B1 showed comparable results, indicating transformers could be viable alternatives	54.5% (M)
Xu Wang [30] 2019	157, 1, RP	CT	HCR + ML (DT, SVM, KNN, RF)	NM	Semi‐automatic (RadiAntViewer software), Thresholding and growing (solitary), hulls and chain code (juxta‐pleural), distance and clustering (vascularized)	NM	PCA	RF Acc 84.7%, AUC 0.88, Sens 0.767, Spec 0.867	Prognostic recurrence analysis using CT features	The Random Forest model based on CT grayscale, shape and texture features achieved high accuracy of 84.7% in predicting recurrence, demonstrating the utility of radiomics features for this task	43.7% (M)
Gihyeon Kim [29] 2022	326, 2, RP	CT + Clinical	HCR (Cox PH, RSF) + DLR (CNN)	Neural network trained only with TNM staging	Manual following RTOG 1106 guidelines	Pyradiomics	Univariate Cox regression	Ensemble model (clinical + HCR + DLR) Acc 73.23%, F1‐score 77.79%, AUC 0.77	Develop an Ensemble model (clinical + HCR + DLR) to predict NSCLC recurrence after surgery	The proposed Ensemble model showed better recurrence prediction than models using individual data sources or conventional TNM staging	72.7% (G)
Doohyun Park [40] 2022	185, 2, RFS (3‐Year)	CT(+C) + Clinical	HCR + ML	RF model trained only with clinical features	Semi‐automatic using Watershed algorithm in 3D Slicer 4.9.0	Pyradiomics	Stability test, prognostic relevance test (Wilcoxon rank‐sum), redundancy test	RF (normalised CT clinical). AUC 0.802	Analyse whether CT image normalisation can improve RFS (3‐Year) prediction in NSCLC	More radiomic features were identified after CT normalisation. Prognostic model performance improved significantly after CT normalisation	92.8% (E)
Margarita Kirienko [10] 2018	295, 1, DFS (3‐Year)	PET + CT + Clinical	HCR + SA (Cox PHR)	Clinical predictors only	Semi‐automated (PET VCAR software) and manually adjust VOI placement on CT scans for lesions	LifeX package	Univariate analysis and correlation analysis	Cox model using radiomic signature from CT images (AUC 0.75) (95% CI: 0.65–0.85)	Identify a CT and PET‐based RS to predict DFS in NSCLC patients undergoing surgery	RS from CT, PET, PET/CT images were predictive of DFS and outperformed common clinical predictors like TNM staging in surgically treated NSCLC patients/ Adding clinical predictors (age, sex, histology, stage) to the radiomic models did not improve performance	74% (G)
Ahn [28] 2019	93, 1, DFS (3‐Year)	PET	HCR + ML (RF, NN, NB, LR, SVM) + SA for clinical	Different ML models	FDG PET‐CT—a Semi‐automatic Gradient‐based segmentation method (MIM software)	CGITA an open‐source MATLAB package	Information gain ratio, Gini index, chi‐square statistics for ranking features	RF AUC 0.956, Acc 0.901, F1 score 0.872, precision 0.905, recall 0.842	To assess the prognostic value of 18F‐FDG PET‐based radiomics using a machine learning approach in NSCLC patients	A PET‐based radiomic model with an RF classifier improved performance in predicting recurrence risk in NSCLC patients after curative resection	70.9% (G)
Yoshihisa Shimada [41] 2022	642, 1, RFS (2‐Year) and DFS (2‐Year) and OS	CT + Clinical	HCR + SA (UV Cox PH, MV Cox PH)	NM	Automatic segmentation using AI software (Fujifilm Corporation)	AI software from Fujifilm Corporation	NA	Multivariate Cox model. For solid part volume ratio > 22.9%. Validation set—Sensitivity: 1.00, Specificity: 0.30, NPV: 1.00. Entire cohort—Sensitivity: 0.93, Specificity: 0.40, NPV: 0.98	To explore the ability of CT‐based radiomics coupled with AI to predict early recurrence in patients with clinical stage 0‐IA NSCLC	CT‐based radiomics coupled with AI, especially solid part volume ratio, contributes to the non‐invasive prediction of early recurrence in early‐stage NSCLC patients after surgical resection	66.9% (G)
Sehwa Moon [42] 2020	219, 1, RP and RFS (2‐Year)	CT + Clinical	HCR + ML (RF, SVM, NBC, BA)	T and N stage	NM	Pyradiomics	Cox univariate analysis, removing features with missing values, near‐zero variance, or severely unbalanced distributions	SVM (clinical + T and N stage + radiomic). Acc 77% (± 5.8%) for Recurrence Prediction and 70% (± 3.0%) for RFS (2‐Year), AUC 0.66	To improve the prediction of NSCLC recurrence risk by incorporating clinical information and radiomics from CT images, using an effective feature extraction method (combining PCA and machine learning)	The proposed algorithm SVM (clinical, T and N stage, CT) showed better performance in predicting NSCLC recurrence compared to using only the T and N stage or only radiomic features	42% (M)
Jaryd R. Christie [19] 2021	135, 2, RP	PET + CT + Clinical	HCR + SA (Cox PH)	Stage‐only	CT—Semi‐automatic segmentation (MATLAB). PET—Semi‐automatic gradient‐based segmentation (MIM Software)	Quantitative Image Feature Engine (QIFE)	LASSO	Multivariate model (clinical, CT, PET) Training cohort: C‐index = 0.81 Testing cohort: C‐index = 0.79	Develop a multi‐modality radiomics model incorporating CT, PET and clinical features to improve risk stratification for recurrence over stage alone in NSCLC	The radiomics model outperformed stage alone in predicting recurrence risk, suggesting it could help identify high‐risk patients who may benefit from more aggressive treatment	64.3% (G)
Yuki Sasaki [32] 2022	150, 1, RP	CT(+C)	DCNN (VGG19)	NM	Manual cropping of tumour region (72 × 72 pixels)	NM	NA	Proposed DCNN model (Sens—0.75 Spec—0.87 Acc—0.82 AUC—0.86)	To propose a CAD system to predict postoperative recurrence from preoperative CT images in lung adenocarcinoma patients using DL	The DL model using preoperative CT images could predict postoperative recurrence with high accuracy, suggesting its usefulness for individualised prediction of recurrence risk before treatment	67.8% (G)
Jie Lian [33] 2022	1705, 1, RFS and OS	CT + Clinical	DL (ViT + GraphSAGE model), (ResNet + Graph model)	(1) TNM staging (2) ResNet + Graph model	Manual (3D Slicer)	Pre‐trained ViT	NA	GraphSAGE + ViT (CT + Clinical) OS AUC: 0.785 (testing), 0.695 (external validation) RFS AUC: 0.726 (testing), 0.700 (external validation)	Predict OS and RFS for early‐stage NSCLC patients using a *Transformer and graph neural network* model that integrates *imaging and non‐imaging data*	The Transformer‐Graph model effectively predicted survival in early‐stage NSCLC by combining *ViT imaging features* and *clinical data in a population graph*. Clinical data helped identify high‐risk patients, while ViT features provided additional discriminative power when clinical data was non‐discriminative. The model outperformed TNM staging and a *ResNet imaging* model on internal and external validation sets	89.9% (E)
Tugba Akinci D'Antonoli [43] 2020	124, 1, RP	CT + Clinical	HCR + SA (Cox PH, LR)	TNM staging	Semi‐automatic (Eclipse software)	In‐house Moddicom software	Univariate filtering, fscaret R package for recursive feature elimination	Multimodality radiomic mode (tumour + peritumoral + TNM staging) (AUC = 0.760 for Total recurrence AUC = 0.759 for Distant metastasis AUC = 0.750 for Local recurrence)	To estimate recurrence risk after surgery in NSCLC patients by *radiomics analysis of tumour and peritumoral* lung parenchyma	*Combination* of tumoral and peritumoral RS *with* TNM staging outperformed TNM staging alone for individualised recurrence risk estimation in surgically treated NSCLC patients	87.6% (E)
Peiwen Wang [31] 2024	152, 1, RP	CT + Clinical	HCR + SA (LR, Cox PHR) + ML (SVM, KNN, ETs, RF, XGBoost, LightGBM)	N stage only (Logistic regression)	Manual (3D Slicer)	Pyradiomics	Univariate analysis, Spearman correlation, LASSO	R‐C model (RS + CS) (Training AUC = 0.972, Test AUC = 0.937 Training C‐index = 0.815, Test C‐index = 0.847)	Construct a comprehensive model (radiomics + clinical) to predict postoperative recurrence risk in NSCLC patients	The R‐C model showed better predictive performance than the clinical or radiomics model alone for *stratifying recurrence risk* in operable NSCLC patients after surgery. *Adding* clinical features (N stage) improved model performance over radiomics model alone	80.8% (E)
Jaryd R. Christie [17] 2022	135, 1, PP and RP	PET + CT + Clinical	HCR + ML (RSF)	Stage‐only	CT—Semi‐automatic (MATLAB) FDG PET‐CT Semi‐automatic gradient‐based method [a part of Commercial software (MIM 6.6)]	Pyradiomics	LASSO	Multimodality radiomic model () with stage. Training cohort: Concordance = 0.78Testing cohort: Concordance = 0.76	Develop a multimodal tumour/non‐tumour radiomics + clinical data to improve risk stratification in resectable NSCLC	Multimodality model (stage + CT + PET) for tumour/peritumoral significantly stratified NSCLC patients into low‐ and high‐risk groups for recurrence/progression compared to using stage alone	85.7% (E)

Abbrevations: Acc,Accuracy; CT,Computed Tomography; CS,Clinical Signature; DFS, Disease‐Free Survival; DL,Deep Learning; DLR,Deep Learning Radiomics; DRS,Deep learning Risk Score; ETs,Extra Trees; HCR, Hand‐Crafted Radiomics; LR,Logistic Regression;ML, Machine Learning;NA,Not Available; NM,Not Mentioned; OS, Overall Survival; PCA,Principal Component Analysis;PET, Positron Emission Tomography; PP,Prognosis Prediction; R‐C model,Radiomics‐Clinical model; RF,Random Forest; RFS,Recurrence Free Survival; RP, Recurrence Prediction; RRS,Radiomics Risk Score; RS,Radiomics Signature; SA,Statistical Analysis; Sens,Sensitivity; Spec,Specificity.

Table 3 summarises the distribution of studies based on various strategies for predictive modelling, encompassing data modalities, AI techniques, segmentation methods, feature extraction methods, and feature selection strategies.

**TABLE 3:** Summary of reviewed studies—data modalities, AI models, segmentation, feature extraction, and feature selection methods.

Modelling strategies	Specific method	Number of studies
Data modality	CT	4
	PET	1
	CT + Clinical Data	8
	PET + CT + Clinical Data	3
AI models	Machine learning (radiomics)	5
	Statistical analysis (radiomics)	5
	Deep learning	6
Segmentation methods	Manual segmentation	7
	Semi‐automatic segmentation	7
	Automatic segmentation	1
	Not mentioned	1
Feature extraction methods	Pyradiomics	6
	Deep learning models	3
	LifeX, CGITA, QIFE, etc.	5
	Not mentioned	2
Feature selection methods	LASSO	4
	Univariate and multivariate methods	8
	Not applicable	4

### 3.3 Quality Assessment Results

The scores were calculated as a percentage, with higher METRICS scores indicating better adherence to methodological standards and higher quality of the radiomics research. As shown in Table S1, the quality assessment of the studies revealed that six studies were scored as excellent, four as moderate, and six as good quality. Items that did not apply to a particular study were marked as “no.” For items that were applied in the study or implicitly covered in the articles but not explicitly mentioned, the score was marked as “yes.”

### 3.4 Radiomics and Artificial Intelligence Models

Four studies used hand‐crafted radiomics (HCR) features and then analysed them with statistical analysis (SA) (4/16, 25%) [10, 19, 41, 43], six articles extracted HCR features and then analysed them with different ML algorithms (6/16, 37.5%) [17, 28, 30, 31, 40, 42] one study used both HCR and DL radiomics (DLR) features and then assessed the correlation between these two sets of features to build the risk prediction models (RRS or DRS) [40], another study used both extracted DLR features as well as end‐to‐end CNN model, along with HCR features and clinical data, in their proposed ensemble approach [29]. Four studies applied different state‐of‐the‐art DL models to predict the patient outcome (4/16, 25%) [7, 23, 46, 47], with two of them only relying on CT images [23, 44] and the other two entering both CT images and clinical data to the DL models [7, 33].

### 3.5 Model Building Strategies and the Baseline Model

#### 3.5.1 Segmentation

Regarding the studies included in this review, the number of studies that employed manual segmentation (7/16, 43.75%) [7, 23, 29, 32, 33, 34, 40] and semi‐automatic segmentation (7/16, 43.75%) [10, 17, 19, 28, 30, 40, 43] were similar. One study utilised automatic segmentation (1/16, 6.25%) [41], while another (1/16, 6.25%) did not provide information about the segmentation approach employed [42].

#### 3.5.2 Feature Extraction

In this review, the method most commonly used for feature extraction by the studies was Pyradiomics. (6/16, 37.5%) [17, 29, 31, 40, 42, 45]. Three studies (3/16, 18.75%) used DL models for feature extraction [7, 23, 33], and other five studies (5/16, 31.25%) [10, 19, 28, 41, 43] employed different techniques for feature extraction, which are provided in Table 2. Moreover, in two studies [30, 32], the feature extraction process was either not applicable or not explicitly mentioned.

#### 3.5.3 Feature Selection

The most employed technique for feature selection among the studies investigated in this review was LASSO (4/16, 25%) [17, 19, 31, 45]. Feature selection was not applicable for three studies since they applied DL models [32, 33, 41]. One study did not mention the feature selection method [23], and half of the studies (8/16, 50%) [7, 10, 28, 29, 30, 40, 42, 43] used different feature selection methods provided in Table 2.

#### 3.5.4 Baseline Model

Establishing and comparing models against a baseline is crucial for evaluating the effectiveness and potential improvements offered by more advanced or complex models [44]. A majority of the studies (9/16, 56.25%) employed clinical staging as a baseline model to assess the comparative performance of their proposed models [10, 17, 19, 29, 31, 33, 40, 42, 43]. However, three studies did not mention the use of any baseline model for comparison (3/16, 18.75%) [30, 32, 41], while four studies (4/16, 25%) [7, 23, 28, 45] compared the predictive capabilities of the various models they developed against each other.

#### 3.5.5 Number of Cohorts and Sample Size

Except for three multicentre articles [19, 29, 40], all other studies were single‐centre. Regarding sample size, the largest study had 1705 patients [33]. The other fifteen studies included an average of 235 patients, ranging from a minimum of 93 [28] to a maximum of 642 [41]. Kirienko et al. [10] who employed a cohort of 295 patients to study PET + CT + clinical data, studied with the largest dataset using these three modalities. Almost all the articles focused on the early stages of “NSCLC” and “surgery” as a curative treatment, among which two articles studied only a subtype of NSCLC, the early stage of lung adenocarcinoma [32, 45], and Moon et al. and Kirienko et al. studied NSCLC across different stages [10, 42].

#### 3.5.6 Prognosis Outcome

The following definitions of various prognostic outcomes are provided: disease‐free survival (DFS), defined as the time span between the surgery date and the date of recurrence, second lung cancer, death from any cause, or the last known date the patient was alive; recurrence‐free survival (RFS), defined as the interval between the date of surgery and the date of recurrence, date of death from any cause, or date on which the patient was last known to be alive [41]; PFS defined as the period from the beginning of treatment until either tumour progression or death from any cause, with patients who are lost to follow‐up being excluded from the analysis [46]; overall survival (OS), defined as the interval between the date of surgery and the date of death from any cause or the date on which the patient was last known to be alive [41]; and recurrence, categorised as local, regional, or distant for curable lung cancer (stages I–IIIA) [47]. Regarding the primary outcome measures, eight studies [7, 19, 23, 29, 31, 32, 33, 44] focused on recurrence prediction (RP), while two others [10, 28] concentrated on disease‐free survival (DFS) within 3‐years after surgery. One study [40] measured recurrence‐free survival (RFS) within 3 years following the operation. Another study [41] investigated three outcomes: RFS (2‐years), DFS (2‐years), and overall survival (OS). Additionally, two studies reported prognosis prediction (PP) along with RP [17] and OS [45]. One study [42] utilised both RP and RFS within 2 years, while another [33] predicted both 5‐year RFS and OS.

#### 3.5.7 External Validation

In terms of external validation, only three studies (3/16, 18.75%) applied external validation [33, 40, 45]. Two of them [32, 39] utilised the public dataset as external validation, while another [30] employed a private dataset.

## 4 Discussion

This review effectively fulfilled its primary objectives by providing a comprehensive analysis of studies utilising AI techniques to predict postoperative recurrence risk in early‐stage NSCLC. The findings underscore the potential of radiomics and deep learning models in recurrence prediction. Multimodal approaches integrating CT, PET, and clinical data consistently outperform single‐modality models, with convolutional neural networks demonstrating notable effectiveness in this domain [7, 29].

### 4.1 Radiomics Studies

#### 4.1.1 CT + Clinical Data

A growing body of research has showcased the potential of radiomics features extracted from computed tomography (CT) images in predicting the postoperative risk of recurrence in patients diagnosed with early‐stage non‐small cell lung cancer (NSCLC). Notably, the studies conducted by Wang et al. [31] and D'Antonoli et al. [43] demonstrated that radiomics features, particularly those pertaining to texture analysis, exhibited significant predictive value for assessing the risk of recurrence, as evidenced by the area under the receiver operating characteristic curve (AUC) values of 0.916 and 0.74, respectively. Furthermore, the integration of these radiomics features with clinicopathological data, such as tumour‐node‐metastasis (TNM) staging, resulted in enhanced predictive performance, with AUC values increasing to 0.937 and 0.760, respectively. These findings underscore the superiority of the combined approach, incorporating both radiomics and clinical data, compared to relying solely on clinicopathological factors, which yielded lower AUC values of 0.788 and 0.24, respectively, in the aforementioned studies.

The superior predictive performance achieved by integrating radiomics features and clinicopathological data was further corroborated by the studies conducted by Park et al. [40] and Moon et al. [42] In the former study, the AUC for 3‐year RFS was found to be 0.802 for the combined clinical and radiomics model (with normalised CT features). This result outperformed the AUC of 0.69 obtained from the clinical‐only model, underscoring the added value of radiomics in enhancing prognostic accuracy. Similarly, the study carried out by Moon et al. [42] reported AUC values of 0.66 and 0.74 for recurrence prediction and 2‐year RFS, respectively, when employing a combined radiomics and clinical model. However, it is noteworthy that in this particular study, the difference in performance between the integrated model and the clinical‐only model did not reach statistical significance. The authors deduced that the inability of the radiomics‐only model to achieve comparable results could be attributed to the ineffectiveness of the principal component analysis (PCA) technique in adequately reducing the dimensionality of the extracted radiomics features.

#### 4.1.2 PET + CT + Clinical Data

Some studies employed PET images to build radiomics‐based DFS (3‐year) models. The studies by Ahn et al. [28] and Kirienko et al. [10] specifically utilised texture features extracted from PET images to develop DFS prediction models. Ahn et al. [28] demonstrated the prognostic value of contrast and busyness texture features from the neighbourhood grey‐tone difference matrix (NGDM), which outperformed conventional PET metrics such as metabolic tumour volume (MTV), total lesion glycolysis (TLG), or standardised uptake value (SUV). Their random forest model achieved an impressive AUC of 0.956, surpassing other machine learning models investigated in their study. Kirienko et al. [10] reported AUC values for various combinations of modalities. For PET images alone, the radiomics signature yielded an AUC of 0.66 for predicting 3‐year DFS, outperforming the clinical model (AUC = 0.58) and the combined PET and clinical model (AUC = 0.64).

When considering CT images, the radiomics signature, clinical data, and CT + clinical data models achieved AUC values of 0.62, 0.58, and 0.61, respectively. In this study, however, adding clinical predictors such as age, sex, histology, and stage to the individual radiomic modality of PET‐only and CT‐only did not improve the performance. Still, for the multimodality radiomics model of PET + CT images, the AUC values were measured as 0.62 for the radiomics signature, 0.61 for clinical data, and 0.65 for the PET + CT + clinical model. These results further support that the combination of multimodal imaging data and clinical information has shown promising results in improving prognostic accuracy compared to models based on a single modality or clinical data alone. Likewise, the study by Christie et al. [17] further highlighted the importance of incorporating both PET and CT data into a multimodal radiomics model to enhance predictive accuracy. While they did not investigate a radiomics‐only model, their findings aligned with previous literature demonstrating the association between tumour heterogeneity in CT and PET and progression and treatment failure. Christie et al. [17] achieved a concordance index of 0.60 for the stage‐only model and an improved concordance index of 0.76 for the PET + CT + clinical model.

Performance comparisons across studies reveal a diverse landscape of methodologies and outcomes. For models combining CT and clinical data, AUC values ranged from 0.66 to 0.937 [7, 29, 31, 33, 40, 41, 42, 43] while models incorporating PET, CT, and clinical data achieved AUC and C‐indices of 0.65, 0.76 and 0.79, respectively [10, 17, 19]. Notably, some single‐modality studies reported exceptionally high‐performance metrics, such as Wang et al. [31] (AUC = 0.937) and Ahn et al. [28](AUC = 0.956). However, it is crucial to interpret these results cautiously, considering the variations in datasets, methodologies, and evaluation protocols across studies.

The apparent contradiction where some single‐modality studies outperform multimodality approaches in cross‐study comparisons can be attributed to several factors. These include differences in dataset characteristics, methodological variations in preprocessing and feature extraction, potential overfitting in highly optimised single‐modality approaches, and disparities in evaluation metrics and protocols. Moreover, publication bias may skew perceptions of typical performance levels. Despite these challenges in direct cross‐study comparisons, multimodality approaches often demonstrate more consistent improvement over single‐modality techniques within individual studies [17, 19, 29]. This suggests that combining multiple imaging modalities (e.g., PET and CT) with clinical data offers the potential for more comprehensive and robust analysis [48].

### 4.2 Deep Learning Approaches

DL models, particularly CNNs, have emerged as powerful tools for extracting relevant features directly from medical images without the need for explicit feature engineering [49]. This automatic feature extraction capability is a significant advantage over traditional radiomics approaches, potentially capturing subtle patterns that might be overlooked in handcrafted feature extraction methods [34]. Sasaki et al. [32] developed an end‐to‐end DCNN based on VGG19 that could predict the postoperative recurrence risk of primary lung adenocarcinoma with high accuracy (0.82) using preoperative CT images. This study demonstrates the potential of deep learning to derive prognostic information directly from raw imaging data, without the need for intermediate feature extraction steps.

Transfer learning approaches, where pre‐trained models are fine‐tuned on the NSCLC dataset, have been explored to overcome the challenge of limited data availability required for DL models [50]. This technique leverages knowledge gained from large, general image datasets to improve performance on specific medical imaging tasks, even with limited sample sizes [51].

Bove et al. [7] and Fanizzi et al. [23] evaluated the performance of different pre‐trained CNNs and transformer architectures (Vision Transformers, Pyramid Vision Transformers, and Swin Transformers) in predicting NSCLC recurrence, with CNNs generally outperforming the transformer models. This suggests that while transformer models have shown promise in natural language processing and some computer vision tasks, CNNs may still have an edge in medical image analysis for NSCLC recurrence prediction.

AlexNet CNN with AUC = 0.83 in the study of Bove et al. [7] and InceptionV3 CNN with AUC = 0.91 in the study of Fanizzi et al. [23] were the best‐performing DL models in predicting postoperative recurrence of NSCLC. These high AUC values are impressive, especially considering that they were achieved using single‐modality imaging data. However, it is important to note that direct comparisons between studies should be made cautiously due to differences in datasets, preprocessing techniques, and evaluation protocols.

Another study conducted by Lian et al. [33] introduced a novel Transformer‐Graph model that combined imaging features from a transformer network with clinical data using a graph neural network, outperforming conventional models in predicting overall survival and recurrence‐free survival (5‐year) in early‐stage NSCLC patients. Their combined model [GraphSAGE + ViT (CT + Clinical)] outperformed TNM staging and ResNet imaging model alone on both internal and external validation sets [RFS AUC: 0.726 (testing), 0.700 (external validation)]. This study represents a significant advancement in integrated approaches, leveraging state‐of‐the‐art deep learning architectures to effectively combine imaging and clinical data. Despite these promising results, deep learning approaches face challenges in clinical adoption. The “black box” nature of many DL models can hinder interpretability, which is crucial for clinical decision‐making [52]. Additionally, the performance of these models can be sensitive to variations in imaging protocols and equipment, potentially limiting their generalizability across different clinical settings [53].

### 4.3 Integrated Approaches

To harness the strengths of radiomics and deep learning, several studies have proposed integrated models combining handcrafted radiomics features, deep learning‐based features, and clinical data. These models aim to provide a more comprehensive tumour representation, improving prediction accuracy and robustness [54]. Cho et al. [45] proposed a radiomics‐guided deep learning approach that combined pretrained deep learning models with radiomics features, improving interpretability and reducing sample size requirements. Despite not incorporating clinical data, this study achieved a C‐index of 0.7696 by the Yolo model, showing promise for lung adenocarcinoma prognosis. This approach showcases how the integration of radiomics and deep learning can potentially overcome some limitations of each method used individually. Similarly, Kim et al. [29] developed an ensemble model that integrated handcrafted radiomics, deep learning‐based radiomics (CNN), and clinical data, achieving better performance (Acc 73.23%) than each of the individual models. This study highlights the potential synergy between different types of features and data modalities in improving predictive accuracy.

The performance of these integrated models, while not always achieving the highest absolute AUC values seen in some single‐modality studies, demonstrates more consistent improvement over baseline models and potentially offers greater robustness. However, these approaches come with challenges, such as higher computational demands, a greater risk of overfitting, and the need for high‐quality data from multiple sources [55]. Future research in this area should focus on standardised comparisons of these integrated approaches against single‐modality methods across diverse datasets, enhance model interpretability, and explore the biological basis of features and predictions. Prospective clinical studies are essential to validate their real‐world performance and utility in predicting NSCLC recurrence.

### 4.4 Challenges and Limitations of Included Studies

Despite advancements in radiomics and AI models for predicting postoperative recurrence in early‐stage NSCLC, several challenges persist. Small sample sizes in many studies limit generalizability and increase overfitting risk, especially for high‐dimensional radiomics and deep‐learning models [56, 57, 58, 59]. Variability in imaging quality across institutions further impacts model performance and reproducibility, emphasising the need for larger, standardised datasets [56, 60, 61]. Another major limitation is the lack of external validation using independent datasets, which restricts assessments of model reliability across diverse populations and imaging protocols [62, 63]. Without robust external validation, the clinical applicability of these models remains uncertain. Additionally, the predominance of retrospective study designs introduces biases, such as selection and information bias, that undermine the reliability of findings [64]. Prospective studies are needed to evaluate predictive power more accurately.

The interpretability of AI models also poses challenges. Many models lack transparency regarding the biological mechanisms underlying their predictions, reducing clinician trust and hindering adoption [34, 65]. Enhancing model interpretability and linking features to biological insights are essential for clinical integration. The integration of diverse data sources, including multiple imaging modalities (e.g., CT, PET) and clinical data, presents significant challenges [66]. These include data harmonisation across different institutions and imaging protocols, optimal feature selection from high‐dimensional data, and the development of effective multimodality fusion techniques [67]. Balancing the complexity of these integrated models with their generalizability and computational efficiency remains an ongoing challenge [68].

The lack of standardised methods in data preprocessing, feature extraction, model development, and performance evaluation hinders reproducibility and makes direct comparisons between studies challenging, limiting the establishment of best practices in the field [69, 70]. Additionally, translating AI models into clinical practice faces barriers such as integration into existing workflows, ensuring real‐time processing capabilities and demonstrating tangible improvements in patient outcomes through prospective clinical trials [71].

One limitation of this study is its design as a narrative review rather than a systematic review. Future work should aim to conduct a systematic review to achieve a more exhaustive and standardised evaluation of the literature. Another limitation is the selection of databases—Google Scholar, PubMed, and Scopus—which, while broadly relevant, may have excluded specialised AI repositories such as IEEE Xplore and ACM Digital Library. Expanding the database selection in future reviews could provide a more comprehensive assessment of AI applications in oncology.

## 5 Conclusions

This review critically analysed literature from 2018 to 2024 on AI techniques for predicting postoperative recurrence in early‐stage NSCLC. The findings highlight the potential of radiomics, deep learning models, and multimodal systems to enhance prognostic accuracy. Approaches combining imaging modalities (CT and PET) with clinical data often outperform single‐modality or clinical‐only models. Convolutional neural networks have shown notable success in extracting features from medical images. However, fully leveraging AI requires addressing challenges such as larger multicentre studies, external validation, improved interpretability, and standardised methodologies. Advancing data integration, multimodality fusion, and interdisciplinary collaboration are essential for creating clinically actionable AI models that improve patient stratification and personalised treatment strategies, ultimately enhancing outcomes and cancer management.

## Conflicts of Interest

The authors declare no conflicts of interest.

## Supporting information


Data S1.

## Data Availability

Data sharing not applicable to this article as no datasets were generated or analysed during the current study.
