# Supplementary material for: A Comprehensive Review on the Application of Artificial Intelligence for Predicting Postsurgical Recurrence Risk in Early‐Stage Non‐Small Cell Lung Cancer Using Computed Tomography, Positron Emission Tomography, and Clinical Data
Source: J Med Radiat Sci. 2025 Jan 23;72(3):280–96. doi: 10.1002/jmrs.860 (PMC12420675; doi:10.1002/jmrs.860)
Supplement: Supplementary file 1 — Data S1. [file JMRS-72-280-s001.docx]

**Supporting Information (Table S1)**

**Table S1.** Metrics Scoring of included studies.

| ***Items*** | ***Weights*** | ***Ho Cho***^41^ | ***Bove***^7^ | ***Fanizzi***^23^ | ***Xu Wang***^31^ | ***Kim***^29^ | ***Park***^42^ | ***Kirienko***^30^ | ***Christie 2022***^17^ | ***Ahn***^28^ | ***Shimada***^43^ | ***Moon***^44^ | ***Christie 2021***^19^ | ***Sasaki***^33^ | ***Lian***^34^ | ***D'Antonoli***^45^ | ***Peiwen Wang***^32^ |
| --- | --- | --- | --- | --- | --- | --- | --- | --- | --- | --- | --- | --- | --- | --- | --- | --- | --- |
| 1 | 0.0368 | yes | yes | yes | yes | yes | yes | yes | yes | yes | yes | yes | yes | yes | yes | yes | yes |
| 2 | 0.0735 | yes | no | no | no | yes | yes | yes | yes | yes | yes | no | no | yes | yes | yes | yes |
| 3 | 0.0919 | yes | yes | yes | yes | yes | yes | yes | yes | yes | yes | yes | yes | yes | yes | yes | yes |
| 4 | 0.0438 | yes | no | no | no | yes | yes | no | yes | no | no | no | yes | no | no | no | no |
| 5 | 0.0292 | yes | yes | yes | yes | yes | yes | yes | yes | yes | yes | yes | yes | yes | yes | yes | yes |
| 6 | 0.0438 | yes | yes | yes | no | no | yes | yes | yes | yes | yes | no | yes | yes | yes | yes | yes |
| 7 | 0.0292 | yes | yes | yes | no | yes | yes | yes | yes | yes | yes | yes | yes | yes | yes | yes | yes |
| 8 | 0.0337 | *yes* | no | no | yes | yes | yes | yes | yes | yes | yes | no | yes | yes | yes | yes | yes |
| 9 | 0.0225 | n/a | n/a | n/a | n/a | n/a | n/a | n/a | n/a | n/a | no | n/a | n/a | n/a | n/a | n/a | n/a |
| 10 | 0.0112 | no | no | no | no | no | yes | no | yes | no | yes | no | yes | no | yes | yes | no |
| 11 | 0.0622 | yes | no | yes | yes | yes | yes | yes | yes | yes | yes | no | yes | yes | yes | yes | yes |
| 12 | 0.0311 | yes | n/a | no | no | yes | yes | yes | yes | yes | yes | yes | no | n/a | n/a | yes | no |
| 13 | 0.0415 | yes | yes | yes | yes | yes | yes | yes | yes | yes | yes | yes | yes | yes | yes | yes | yes |
| 14 | 0.0200 | yes | yes | yes | no | no | yes | no | yes | no | no | yes | no | n/a | n/a | yes | yes |
| 15 | 0.0200 | yes | yes | yes | yes | yes | yes | yes | yes | yes | no | yes | no | n/a | n/a | yes | yes |
| 16 | 0.0300 | yes | no | no | yes | no | yes | no | yes | yes | no | no | no | n/a | n/a | yes | yes |
| 17 | 0.0200 | n/a | n/a | n/a | n/a | no | n/a | n/a | n/a | n/a | no | n/a | n/a | yes | yes | n/a | n/a |
| 18 | 0.0599 | yes | yes | yes | no | no | yes | yes | yes | yes | yes | no | yes | yes | yes | yes | yes |
| 19 | 0.0300 | no | no | no | no | no | yes | yes | yes | no | no | no | no | no | yes | yes | yes |
| 20 | 0.0352 | yes | yes | yes | yes | yes | yes | yes | yes | yes | yes | yes | yes | yes | yes | yes | yes |
| 21 | 0.0234 | yes | no | no | no | yes | yes | yes | yes | yes | yes | no | yes | no | yes | yes | yes |
| 22 | 0.0176 | no | no | no | no | no | no | no | no | no | no | no | no | no | no | yes | yes |
| 23 | 0.0117 | no | no | no | no | no | no | no | no | no | no | no | no | no | no | yes | yes |
| 24 | 0.0293 | no | no | no | no | yes | yes | yes | yes | no | yes | yes | yes | no | yes | yes | yes |
| 25 | 0.0176 | yes | no | no | no | yes | no | no | no | no | no | no | no | no | yes | yes | yes |
| 26 | 0.0375 | yes | yes | yes | yes | yes | yes | yes | yes | yes | yes | yes | yes | yes | yes | yes | yes |
| 27 | 0.0749 | yes | no | no | no | yes | yes | no | no | no | no | no | no | no | yes | no | no |
| 28 | 0.0075 | no | yes | yes | no | no | no | no | no | no | no | no | yes | no | no | yes | no |
| 29 | 0.0075 | yes | yes | yes | no | no | no | no | yes | no | no | no | no | no | yes | yes | no |
| 30 | 0.0075 | yes | yes | no | no | no | no | no | no | no | no | no | no | no | no | yes | no |
| Q.C |  | E | M | M | M | G | E | G | E | G | G | M | G | G | E | E | E |

***METhodological RadiomICs Score (METRICS).* Q.C**: Quality Category. **E**: Excellent, **M**: Moderate, **G**: Good. **n/a**: Not Applicable. **Item#1**: Adherence to radiomics and/or machine learning-specific checklists or guidelines. **Item#2**: Eligibility criteria that describe a representative study population. **Item#3**: High-quality reference standard with a clear definition. **Item#4**: Multi-center. **Item#5**: Clinical translatability of the imaging data source for radiomics analysis. **Item#6**: Imaging protocol with acquisition parameters. **Item#7**: The interval between imaging used and reference standard. **Item#8**: Transparent description of segmentation methodology. **Item#9**: Formal evaluation of fully automated segmentation. **Item#10**: Test set segmentation masks produced by a single reader or automated tool. **Item#11**: Appropriate use of image preprocessing techniques with transparent description. **Item#12**: Use of standardized feature extraction software. **Item#13**: Transparent reporting of feature extraction parameters, otherwise providing a default configuration statement. **Item#14**: Removal of non-robust features. **Item#15**: Removal of redundant features. **Item#16**: Appropriateness of dimensionality compared to data size. **Item#17**: Robustness assessment of end-to-end deep learning pipelines. **Item#18**: Proper data partitioning process. **Item#19**: Handling of confounding factors. **Item#20**: Use of appropriate performance evaluation metrics for task. **Item#21**: Consideration of uncertainty. **Item#22**: Calibration assessment. **Item#23**: Use of uni-parametric imaging or proof of its inferiority. **Item#24**: Comparison with a non-radiomic approach or proof of added clinical value. **Item#25**: Comparison with simple or classical statistical models. **Item#26**: Internal testing. **Item#27**: External testing. **Item#28**: Data availability. **Item#29**: Code availability. **Item#30**: Model availability.
